# Supplementary material for: Fungi Can Be More Effective than Bacteria for the Bioremediation of Marine Sediments Highly Contaminated with Heavy Metals
Source: Microorganisms. 2022 May 9;10(5):993. doi: 10.3390/microorganisms10050993 (PMC9145406; doi:10.3390/microorganisms10050993)
Supplement: Supplementary file 1 [file microorganisms-10-00993-s001.zip › microorganisms-1663523-supplementary.pdf]

Supplementary materials

Fungi can be more effective than bacteria for the bioremediation of marine sediments highly contaminated with heavy metals

Filippo Dell'Anno, Eugenio Rastelli, Emanuela Buschi, Giulio Barone, Francesca Beolchini and Antonio Dell'Anno

This file includes:

Supplementary Methods

Supplementary Figure 1

Supplementary references

Supplementary Methods

Bacterial and fungal counts under epifluorescence microscopy

For the determination of the bacterial abundance, sediment aliquots were treated with pyrophosphate (5 mM final concentration) and ultrasound (three times for 1 min) [107]. Sub-samples of 1 mL were properly diluted, stained with Acridine Orange (0.01% final concentration), filtered on Nuclepore black 0.2 µm filters, then analysed by epifluorescence microscopy (Zeiss Axioplan microscope, 50-W lamp, 1000 × magnification). A minimum of 20 fields and of 400 cells were counted for each slide, and counts were normalized to sediment dry weight after desiccation (60 °C, 24 h). For the determination of fungal biomass, we used fluorescence in-situ hybridisation (FISH) coupled with Calcofluor white staining following the procedure previously described [108]. Briefly, sediment aliquots were fixed for 1 h in 0.2-µm prefiltered and buffered formaldehyde solution (2% vol/vol). After fixation, samples were centrifuged twice to remove formaldehyde residues and resuspended in PBS. Then, samples were treated using 4 ml of a mix containing EDTA, Tween 80, sodium-pyrophosphate, methanol and ultrasounds treatment to separate fungi from the sediment matrix. After centrifugation, sediment samples were washed twice with PBS buffer and then with with increasing concentrations of ethanol (50, 80 and 96%, for 3 min each). The sample was then suspended in 500 µl hybridisation buffer containing 0.9M NaCl, 0.01% w/v SDS, 20mM Tris–HCl (pH 7.2), 30 %v/v formamide and 1 µM of Cy3-labeled probe PF2 (5′-CTCTGGCTTCACCCTATTC-3′) [109], then incubated for 3 h at 46 °C in the dark. Samples were then transferred in sterile tubes containing pre-warmed washing buffer (20mM Tris–HCl pH 8.0, 0.01% w/v SDS, 5mM EDTA, 0.112M NaCl) and incubated for 30 min at 48 °C. After centrifugation and resuspension of the sediment samples with 0.2 µm pre-filtered water, aliquots were filtered on Nuclepore black 0.2 µm filters. Filters were then stained with 0.5mM Calcofluor white and incubated in the dark for 5 min. Subsequently, filters were washed with 0.2-µm pre-filtered water and analysed under epifluorescence microscopy. The average width and cumulative length of each identified fungal structure was measured under epifluorescence microscopy, then converted to a cylinder with halfspheres at ends, and the biovolume was converted into fungal biomass according to the equation by Menden-Deuer and Lessard [110]: Biomass (pg C) = 0.216 × biovolume (μm^3) $^{0.811}$

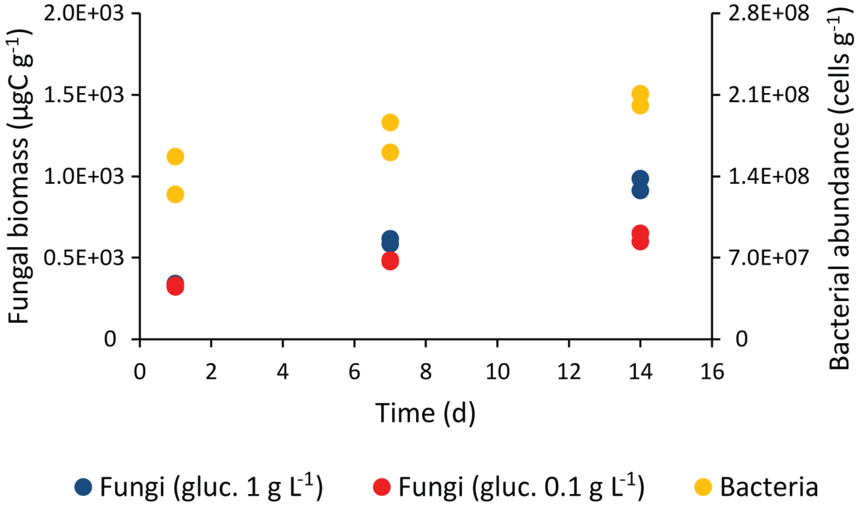

Supplementary Figure S1. Bacterial and fungal abundance in preliminary tests. Reported are the values of fungal biomass (primary y axis) and of bacterial cell counts (secondary y axis) over time in the preliminary experimental systems set up before starting the bioremediation study, to test microbial growth under the test conditions used in this work. Incubation tests were set up at 12.5% w/v (weight of the dry sediment to final volume) in autoclaved 250 mL Pyrex flasks, with 150 mL final volume. For bacterial tests, 15 mL of chemo-autotrophic Fe/S oxidizing bacteria and chemo-heterotrophic bacteria at a concentration of 1.5–2 × 10^8 cells mL^{-1} were inoculated at T0 days. For fungal tests, 10 mg of fungal biomass was added to each microcosm at T0 days, comparing two different glucose concentrations (1 or 0.1 g L^{-1}) in order to assess the growth of the tested marine fungal strains under the more oligotrophic conditions optimal for bacterial growth. For bacteria, the standard glucose concentration of 0.1 g L^{-1} was always used, as previously optimized [10]. All flasks for these preliminary tests were set up in duplicate and kept at constant temperature of 20°C on a rotary shaker (150 rpm) (Stuart orbital incubator S510).

Supplementary references

107. Danovaro, R.; Manini, E.; Dell'Anno, A. Higher abundance of bacteria than of viruses in deep Mediterranean sediments. *Appl. Environ. Microbiol.* **2002**, *68*, 1468-1472.

108. Barone, G.; Rastelli, E.; Corinaldesi, C.; Tangherlini, M.; Danovaro, R.; Dell'Anno, A. Benthic deep-sea fungi in submarine canyons of the Mediterranean Sea. *Progr. Oceanogr.* **2018**, *168*, 57-64.

109. Kempf, V.A.J.; Trebesius, K.; Autenrieth, I.B. Fluorescent in situ hybridization allows rapid identification of microorganisms in blood cultures. *J. Clin. Microbiol*. **2000**, 38, 830–838.

110. Menden-Deuer, S.; Lessard, E.J. Carbon to volume relationships for dinoflagellates, diatoms, and other protist plankton. *Limnol. Oceanogr.* **2000**, 45, 569–579.
